# Do you mind the role of spinal sensory block duration in a crucial endocrine disorder of diabetes mellitus? A prospective observational study

**DOI:** 10.1590/1806-9282.20231727

**Published:** 2024-05-17

**Authors:** Tuna Albayrak, Mucahit Coskun, Ilker Sengul, Aysegul Torun Goktas, Demet Sengul, Mehmet Albayrak, Tuğrul Kesicioglu, Esma Cinar

**Affiliations:** 1 Giresun University, Faculty of Medicine, Department of Anesthesiology and Reanimation – Giresun, Turkey.; 2 Giresun University, Faculty of Medicine, Division of Endocrine Surgery – Giresun, Turkey.; 3 Giresun University, Faculty of Medicine, Department of General Surgery – Giresun, Turkey.; 4 Giresun Education and Research Hospital, Department of Anesthesiology and Reanimation – Giresun, Turkey.; 5 Giresun University, Faculty of Medicine, Department of Pathology – Giresun, Turkey.; 6 Karadeniz Technical University, Faculty of Medicine, Division of Perinatology – Giresun, Turkey.; 7 Karadeniz Technical University, Faculty of Medicine, Department of Obstetrics and Gynecology – Giresun, Turkey.

**Keywords:** Diabetes mellitus, Diabetic neuropathies, Anesthesia, spinal, Pathology, Surgery

## Abstract

**OBJECTIVE:**

Diabetes mellitus, per se, is a global health concern, which is often accompanied by complications such as diabetic neuropathy. This prospective observational study purposed to assess the durations of spinal sensory block and motor blocks in individuals with and without diabetes mellitus who had undergone spinal anesthesia.

**METHODS:**

This study incorporated 80 cases, which were evenly divided into spinal sensory block without diabetes mellitus and spinal sensory block with diabetes mellitus. Various parameters were recorded at different time points, including heart rate, mean arterial blood pressure, SpO_2_, and spinal block characteristics. Notable measures included maximum spinal sensory block onset time, time to reach the 10th thoracic vertebra (T10), maximal spinal sensory block, time for Bromage scores, and block regression while controlling for age-related variations.

**RESULTS:**

Patients in the diabetic group exhibited extended block durations, with significant differences in heart rate noted at specific time points. Regarding the spinal block characteristics, the “maximum onset of SSB” and the “time to reach the T10” were more prolonged in the SSBwDM without significance. Maximum sensory spinal sensory block did not differ. However, some cases in the SSBwDM displayed blocks extending up to the T6. The times to achieve Bromage motor block scores 1–3 were shorter in SSBwDM and lost significance regarding age. Notably, the regression time was longer in SSBwDM, which held significance for both parameters.

**CONCLUSION:**

Diabetic cases commonly encounter prolonged block durations post-subarachnoid intervention, potentially linked to nerve sensitivity, age-related changes, and glycemic control. As such, attenuated local doses for diabetic neuropathic cases may enhance early mobilization, attenuate thromboembolic events, and expedite gastrointestinal recovery.

## INTRODUCTION

Diabetes mellitus (DM), per se, is a chronic metabolic disorder characterized by persistent hyperglycemia, primarily resulting from impaired insulin secretion, resistance to insulin’s peripheral actions, or a combination of both. The chronic elevation of blood sugar levels, in conjunction with other metabolic abnormalities, can inflict harm upon various organ systems, leading to the emergence of debilitating and life-threatening health complications, including microvascular issues such as retinopathy, nephropathy, and neuropathy, as well as macrovascular complications^1^. One of the significant complications of DM is neuropathy, a condition where nerves are damaged, leading to impairment of sensory, motor, and autonomic nerve functions. Peripheral nerve damage is particularly evident in individuals with DM and can result in neuropathy, which may substantially impact daily life activities and overall health. Diabetic neuropathy can manifest as symptoms such as pain, numbness, and tingling, significantly ruining quality of life^2-4^.

In this context, the spinal approach offers advantages such as better hemodynamic control, lower risk of postoperative wound infection, reduced postoperative nausea and vomiting, and potentially faster recovery and mobilization. This study investigates how subclinical peripheral nerve neuropathy in Type 2 DM responds to spinal blocks and aims to compare sensory and motor block durations after the spinal approach and the time elapsed until the first concerning Type 2 DM, whose outcomes could contribute to a better understanding of the use of spinal anesthesia in surgical procedures for diabetic cases.

## METHODS

### Study design

In this prospective study, individuals with Type 2 DM and those without DM who had been scheduled for elective procedures under spinal anesthesia were selected sequentially. The patient eligibility criteria included the age of 18–75 years, a height of 150–180 cm, and a body mass index (BMI) of less than 40 kg/m^2^, while the exclusion criteria were pregnancy, significant vertebral column abnormalities, dehydration, neuropathy history, or contraindications for the spinal method. The primary outcome was the time to reach maximum spinal sensory block (SSB) to develop after the spinal one. A sample size of 40 participants in each was determined using an independent group t-test model with a 20% increase in SSB onset time for the SSBwDM, Cohen’s d effect size of 0.596, 80% power, one-sided confidence interval, and a 5% type 1 error rate.

As such, the study initially included 94 cases, of which 11 were excluded due to unregulated blood sugar, 3 required general anesthesia due to inadequate block, and the remaining 80 were divided into SSB without DM (SSBwoDM) and SSB with DM (SSBwDM). Parameters including heart rate, mean arterial blood pressure, and peripheral oxygen saturation were assessed at seven time points. Demographic data, ASA physical classification, weight, height, BMI, and sex were also collected. The spinal approach was administered through the L3–L4 space, using 15 mg of 0.5% hyperbaric bupivacaine with a 24-G Sprotte needle. Monitoring was conducted from the arrival in the operating room and continued every 5 min for the first 30 post-spinal punctures and then every 15 min until both blocks resolved.

The level of sensory blockade was determined using a cotton swab soaked in alcohol, assessing sensitivity to cold. The motor block level was set concurrently using the Bromage Scale. Patients achieving an SSB below the T6 dermatome and with a Modified Aldrete score exceeding 8 were transferred to the post-anesthesia care unit, and block durations were recorded.

### Statistical analysis

In analyzing the data obtained in the study, IBM-Statistical Package for Social Sciences (IBM-SPSS Inc., Chicago, IL, USA) version 22.0 was used. The normal distribution of the data was assessed using the Shapiro-Wilk test. The continuous variables were expressed as mean and standard deviation or median (25–75 percentile) depending on the distribution, and categorical variables were expressed as numbers and percentages. For the analysis of continuous variables, the independent samples t-test was applied when the parametric test assumptions were met; otherwise, the Mann-Whitney U test was used. The analysis of categorical variables used the chi-square test or Fisher’s exact test. An analysis of variance (ANOVA) was employed for repeated measurements at different times among groups. An analysis of covariance (ANCOVA) was used to control for the age effect. The statistical significance level was set at p<0.05.

## RESULTS

The statistical similarity was observed for weight, height, BMI, and sex when examining the demographic parameters. A significant difference (p<0.001) was observed between the ASA physical categorization score of 2 for all in SSBwDM and 70% for SSBwoDM. Furthermore, SSBwDM mean age was considerably greater (p<0.001) (Table 1). The assessment of SSBwDM revealed that 32 cases (80%) were receiving oral antidiabetic (OAD) treatment, 3 (7.5%) were receiving only insulin, 9 (22.5%) were receiving both OAD and insulin, and 5 (12.5%) were not receiving any medication related to DM. The average HbA1c in SSBwDM was 7.4 (ranging 6.32–8.98). The SSBwDM mean heart rate values were consistently higher when the heart rate parameter (controlled for age) was evaluated. Still, this elevation was significant only for times t5, t6, and t7 (p=0.040, p=0.032, and p=0.003, respectively) (Table 2). Changes in heart rate mean over time (time-group interaction) were significant (p=0.018). The parameters of mean arterial blood pressure and SpO_2_ (both controlled for age) were evaluated, and no difference at all times was recognized (p>0.05). Changes over time in mean arterial blood pressure and SpO_2_ means (time-group interaction) were similar for both (p=0.382 and p=0.158, respectively). The SSB characteristics revealed that the parameters “maximum SSB onset time” and “time to reach T10 for SSB” were more prolonged in cases of SSBwDM. Still, this difference was insignificant (p=0.108 and p=0.366, respectively). Although there was no significant difference in the maximal SSB between them, 22.5% of SSBwDM had the SSB level at T6. In comparison, 12.5% of SSBwoDM had it and 30% of SSBwDM had the SSB level at T7, whereas 25% of SSBwoDM had it and 15% of SSBwDM had the SSB level at T10, and 2.5% in SSBwoDM had it. The times to achieve motor block Bromage scores 1–3 were shorter in SSBwDM. Still, when controlled for age, no significant difference between the groups for these three parameters was detected (p=0.081, p=0.248, and p=0.575, respectively). Herewith, the motor block and SSB regression duration were more prolonged in SSBwDM, which was significant for both parameters (p=0.014 and p<0.001, respectively) (Table 3).

**Table 1:** The demographical and clinical characteristics of the participants.

Characteristics		SSBwoDM n=40	SSBwDM n=40	p-value
Age (years)		47±17	61±12	<0.001
Sex	Male	21 (52.5%)	17 (42.5%)	0.370
	Female	19 (47.5%)	23 (57.5%)	
ASA	1	12 (30%)	0 (0%)	<0.001
	2	28 (70%)	40 (100%)	
Weight (kg)		82±14	84±12	0.487
Height (cm)		170±8	168±9	0.303
BMI (kg/m^2^)		28.3±5.2	29.6±3.9	0.212

Data are mean and standard deviation or number (%). BMI: body mass index, ASA: American Society of Anesthesiologists. Values of p<0.05 are marked in bold.

**Table 2:** The heart rate values in groups according to time.

Time	SSBwoDM n=40	SSBwDM n=40	p-value	Adjusted (age) p-value
T1	76±12	76±11	0.744	0.828
T2	74±12	76±12	0.433	0.464
T3	72±12	73±13	0.741	0.913
T4	67±9	70±13	0.259	0.303
T5	64±9	69±12	**0.015**	**0.040**
T6	63±10	69±12	**0.017**	**0.032**
T7	61±9	69±11	**<0.001**	**0.003**

The data are mean and standard deviation. The values of p<0.05 are marked in bold.

**Table 3:** The spinal block characteristics of the groups.

Characteristics		SSBwoDM n=40	SSBwDM n=40	p-value	Adjusted (age) p-value
Time to achieve maximum sensorial block level (s)		300 (185–365)	300 (245–400)	0.076	0.108
Time to achieve 10th thoracal vertebra sensorial block level (s)		120 (90–190)	125 (90–205)	0.597	0.366
Maximum sensorial block level (thoracal vertebra)	6	5 (12.5%)	9 (22.5%)	0.070	–
	7	10 (25%)	12 (30%)		
	8	23 (57.5%)	13 (32.5%)		
	9	1 (2.5%)	0 (0%)		
	10	1 (2.5%)	6 (15%)		
Time to achieve Bromage score 1 (s)		115 (90–180)	78 (60–120)	**0.009**	0.081
Time to achieve Bromage score 2 (s)		155 (120–240)	120 (95–180)	**0.048**	0.248
Time to achieve Bromage score 3 (s)		270 (205–360)	240 (180–300)	0.476	0.575
Time to regression motor block (min)		197.5 (180–240)	235 (210–240)	**0.007**	**0.014**
Time to regression sensorial block (min)		210 (190–240)	257.5 (230–270)	**<0.001**	**<0.001**

The data are median (25–75 percentiles) or number (%). The values of p<0.05 are marked in bold.

## DISCUSSION

The results of our study confirm that in patients with DM, the dermatomal block following the subarachnoid administration of 0.5% hyperbaric bupivacaine differs from that observed in non-diabetic cases, which is likely due to the increased sensitivity of diabetic nerves to local anesthetics, leading to a longer block duration. Diabetic polyneuropathy, per se, is the result of complex pathophysiological processes primarily triggered by chronic hyperglycemia. Diabetic neuropathy exhibits different responses to regional anesthesia, including a theoretically higher risk of nerve damage due to the initial increase in the nerve’s electric stimulation threshold^5^. Kalichman and Calcutt reported no difference in block duration between diabetic and control animals, while Kroin and Lirk demonstrated extended block durations in diabetic neuropathic animals^6-8^. Furthermore, Kroin et al. delved deeper into ascertaining whether neuropathy or hyperglycemia was responsible for the prolonged block durations, whose findings revealed that long-term glucose control, with concurrent neuropathy attenuation, restored average block duration, whereas acute glucose control or hyperglycemia management did not have the same effect^9^. The effects of diabetes on motor and sensory blocks have been studied in peripheral nerve blocks, but research on the impact of diabetes on spinal blocks is limited^10-16^. Therefore, to the best of our knowledge, this is the first study on this topic in the English-language literature.

One study showed that diabetic patients have more extensive cerebrospinal fluid (CSF) volumes in the brain regions compared with control subjects. This increase in CSF volume, particularly in hypertension and diabetes, did not significantly affect the block level during isobaric spinal anesthesia, so the changes in CSF volume and density in diabetic patients were not suggested to possess a significant clinical impact on spinal blocks^17^. While our study did not find statistical significance in maximum SSB onset time and the time to reach the T10 between both groups, other studies, like Echevarria’s, found that the top block level duration and total regression time were more prolonged in diabetic ones. However, it is essential to note that their study used epinephrine and bupivacaine in their SSB, which is known to prolong block duration due to epinephrine’s vasoconstrictive effect^17^.

A link between CSF volume and the extent of SSB while using iso- or hyperbaric bupivacaine at the lumbar level and its volume has also been inversely correlated with motor block initiation and regression at L1 and L2^17^. Various studies have indicated that in the elderly, SSB increases compared with younger ones, possibly due to age-related physiological changes leading to a decrease in CSF volume^18^. Herewith, our study revealed that the diabetic group had a higher average age, which may have contributed to these differences. The association between the age and the number of blocked dermatomes demonstrated a direct relationship in the diabetic one. In contrast, it was only observed for the maximum dermatomal block duration in SSBwDM.

Long-term complications of diabetes, both microvascular and macrovascular, are a significant cause of morbidity and mortality, and glycated hemoglobin (HbA1c) plays a crucial role in developing and progressing microvascular complications like diabetic neuropathy^19-25^. In this study, higher HbA1c levels had longer block durations, suggesting that poor glycemic control may contribute to extended durations. Of note, the exact mechanisms behind this phenomenon remain unclear. Still, pharmacodynamic (increased sensitivity of sodium currents) and pharmacokinetic (decreased nerve blood flow leading to prolonged local anesthetic residence) factors have been proposed. Endocrine disorders might experience various changes in the autonomic system. Herein, we have reported a significantly higher heart rate in the diabetic group. At the same time, blood pressure values did not differ between them, possibly due to changes in the autonomic nervous system.

### Limitations

Limitations of the study include a relatively small sample size, single-center nature, heterogeneity among diabetic cases, age differences between groups, and the absence of long-term follow-up data.

## CONCLUSION

Our study provides evidence in support of the idea that diabetic patients tend to experience prolonged block durations after subarachnoid intervention. Of note, several factors could contribute to this phenomenon, including the heightened sensitivity of diabetic nerves to local agents, age-related changes, and potentially the impact of glycemic control on duration. In addition, we suggest the need for lower doses of local agents in diabetic neuropathy might enhance early patient mobilization, attenuate thromboembolic events, and expedite the recovery of gastrointestinal function. This issue merits further investigation.
